# Supplementary material for: Momentarily trapped exciton polaron in two-dimensional lead halide perovskites
Source: Nat Commun. 2021 Mar 3;12:1400. doi: 10.1038/s41467-021-21721-3 (PMC7930248; doi:10.1038/s41467-021-21721-3)
Supplement: Supplementary file 1 — Supplementary Information [file 41467_2021_21721_MOESM1_ESM.pdf]

Supplementary Information

Momentary Trapped Exciton Polaron in Two-dimensional Lead Halide Perovskites

Tao et al.

Figure S1. The PL spectrum of 2 L $CsPbBr_3$ NPs in toluene solution shown in logarithm plot.

Figure S2. The PL spectra of 2 L $CsPbBr_3$ thin film at different temperatures and the vogit fits

Figure S3. The Urbach tails and extracted steepness coefficiences σ at different temperatures

Figure S4. The PL spectrum of 3 L CsPbBr3 NPs in toluene solution at 298K and the vogit fit.

Supplementary Note 1. The relationship between steepness constant σ and exciton-phonon interaction strength

Supplementary Note 2. The critical steepness constant for exciton self-trapping

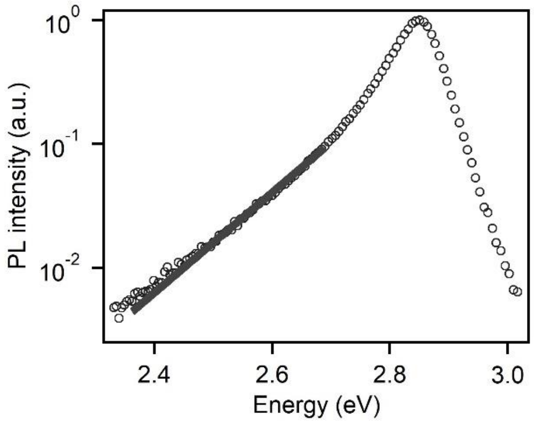

Figure S1. The PL spectrum of 2 L $CsPbBr_3$ NPs in toluene solution shown in logarithm plot.

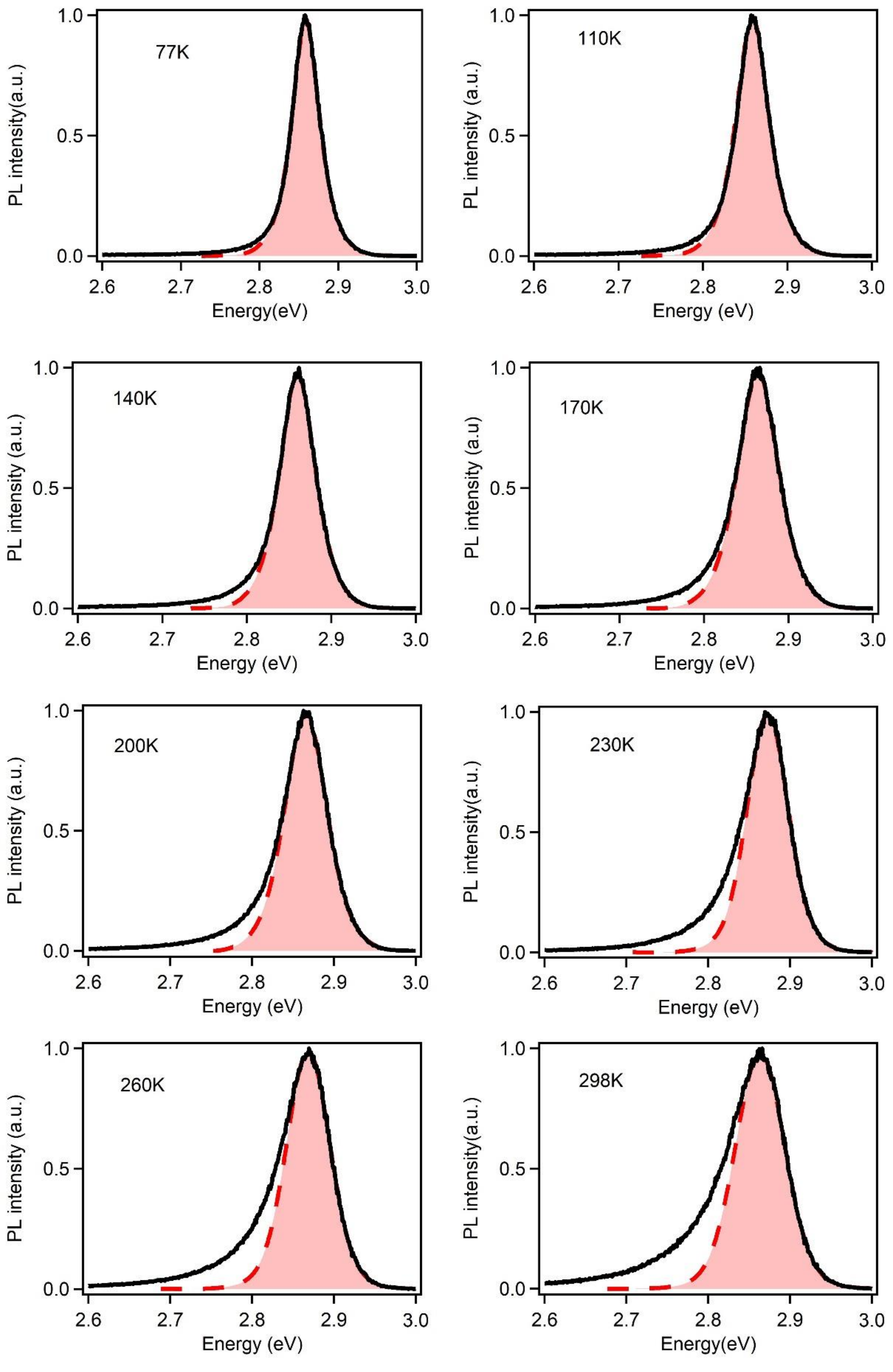

Figure S2. The PL spectra of 2 L $CsPbBr_3$ thin film at different temperatures and the vogit fits

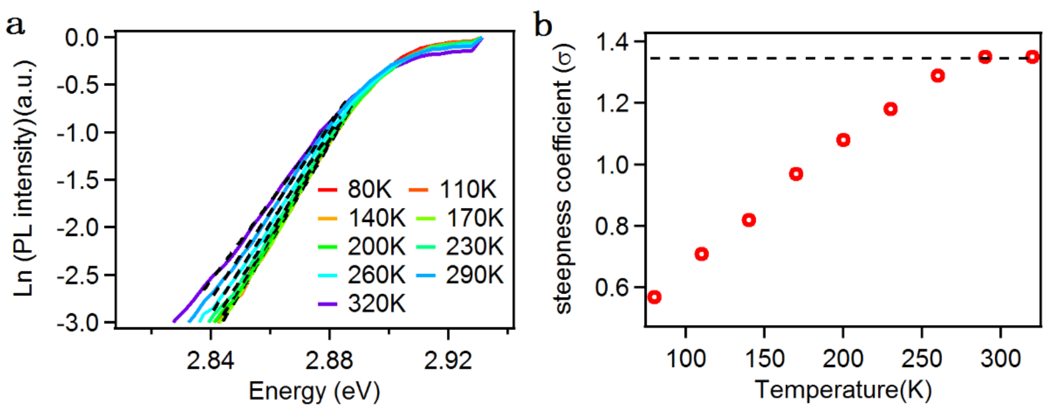

Figure S3. (a) Urbach tail and fitting according to Equ. 4 in main content for 2L CsPbBr3 NPs at different temperature. (b) Extracted steepness coefficient σ at different temperatures, which shows an increase with temperature and reaches a constant (σ_0) at room temperature.

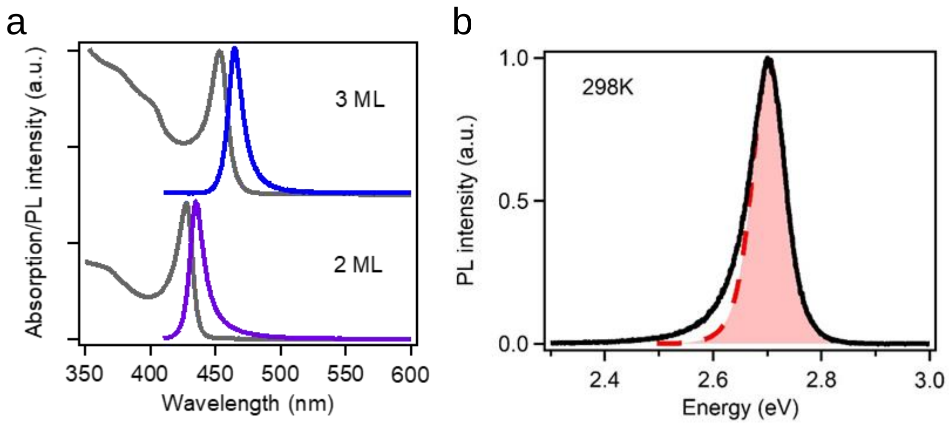

Figure S4. (a) Absorption and PL spectra of our synthesized 2L and 3L perovskite NPs. The absorption/emission peaks are well separated between different thickness due to quantum confinement effect along thickness direction and the peak positions are consistent with literature results. (b) The PL spectrum of 3 L CsPbBr3 NPs in toluene solution at 298 K and the vogit fit.

Supplementary Note 1. The relationship between steepness constant σ and exciton-phonon interaction strength.[1]

The low energy tail of the first exciton absorption peak of a semiconductor follows the empirical rule first discovered by Urbach, which can be expressed as

$$\alpha(E) = \alpha_0 \cdot e^{\frac{-\sigma(E_0 - E)}{k_B T}} \qquad (S1)$$

Where σ is the steepness coefficient of the absorption edge and the temperature dependence of the steepness coefficient σ can be described by Equ S2.

$$\sigma(T) = \sigma_0 \cdot \left(\frac{2kT}{\hbar w_p}\right) \cdot \tanh\frac{\hbar w_p}{2kT} \qquad (S2)$$

It has been considered that the Urbach tail arises from the exciton-phonon interaction since E_0 is usually close to the exciton peak energy. σ increases with temperature and approaches a constant σ_0 at high temperature, as shown by Fig. S3. In this case, the steepness constant σ_0 is independent of temperature and is a material parameter that can quantitively describe the exciton-phonon interaction strength. Smaller σ_0 corresponds to boarder Urbach tail, and hence to larger exciton-phonon interaction strength. For example, σ_0=0.8 for KBr, 1.48 for PbI_2 and 2.17 for CdS, consistent with generally accepted trend of relative exciton-phonon coupling strength. In fact, it has been shown σ_0 is inversely proportional to the strength of exciton-phonon interaction and the detail can be found in Supplementary Note 2. We measured the temperature dependent PLE and plotted the steepness coefficient σ in Figure S3. The trend is consistent with Equ. S2 and indicates σ at room temperature equals to σ_0 for Pb-Br perovskite.

Supplementary Note 2. The critical steepness constant for exciton self-trapping

Here we follow the theory developed by Schreiber and Toyozawa to describe an exciton in the phonon field.[2]They suggested a self-trapped state (the exciton is stabilized by the lattice distortion induced by itself) would form in a deformable lattice. The stability of the self-trapped exciton is decided by the relative energy level between the free exciton and the self-trapped exciton. They introduced a constant g to estimate the exciton-phonon interaction strength which also can describe the nature of the stable exciton state in a particular material. The exciton-phonon coupling constant is defined by

$$g \equiv E_R/B \qquad (S3)$$

where E_R is the lattice relaxation energy, B is the half band width. The emission

spectrum appears narrow near band edge emission when g is much smaller than the critical value g_c while broad, Stokes-shifted emission when g significantly exceeds g_c. When g is near the critical value, both narrow near band edge emission and Stokes-shifted emission can coexist. The $g_c \approx 0.92$ in a three-dimensional lattice and $g_c \approx 0.87$ in a two dimensional lattice[2].

The steepness constant σ mentioned in Supplementary Note 2 is related to the exciton-phonon coupling constant by

$$\sigma = s/g \qquad \text{(S4)}$$

where the dimensionless constant s is called the steepness index, which is independent on the materials constants but only determined by the dimensionality. In three-dimensional and two-dimensional lattice, the steepness index s is a constant, 1.50 and 1.24 respectively. Thus, the critical steepness constant σ for exciton self-trapping is 1.64 and 1.42 for three-dimensional lattice and two-dimensional lattice respectively.

In our case for 2L $CsPbBr_3$ perovskites, the estimated steepness constant σ is 1.48, slightly larger than the critical value 1.42, the self-trapped exciton energy is slighter larger than the free exciton and an energy barrier exists between them. The self-trapped exciton is a meta-stable state.

Supplementary References

1. Sumi, H.; Toyozawa, Y., Urbach-Martienssen Rule and Exciton Trapped Momentarily by Lattice Vibrations. Journal of the Physical Society of Japan **1971,** 31 (2), 342-358.
2. Schreiber, M.; Toyozawa, Y., Numerical Experiments on the Absorption Lineshape of the Exciton under Lattice Vibrations. III. The Urbach Rule. Journal of the Physical Society of Japan **1982,** 51 (5), 1544-1550.
